# Probability of introducing porcine epidemic diarrhea virus into Danish pig herds by imported spray-dried porcine plasma

**DOI:** 10.1186/s40813-015-0010-1

**Published:** 2015-12-11

**Authors:** Alessandro Foddai, Lisbeth Harm Nielsen, Vibeke Møgelmose, Lis Alban

**Affiliations:** 1 Risk Analysis Group, Department of Food Safety and Veterinary Issues, Danish Agriculture & Food Council, Axeltorv 3, DK-1609 Copenhagen V, Denmark; 2 Section of Epidemiology, Technical University of Denmark, National Veterinary Institute, Bülowsvej 27, DK-1870 Frederiksberg C, Denmark

**Keywords:** Import, Risk assessment, Spray-dried porcine plasma, Stochastic simulation model

## Abstract

**Background:**

Porcine epidemic diarrhea virus (PEDV) has never been reported in Denmark, but it has been found in Europe, Asia and North America. Ultimately, PEDV has been associated with devastating outbreaks in pig farms. We developed a stochastic simulation model to carry out a quantitative risk assessment and to estimate the annual probability (*PPlasma*) of introducing PEDV into the Danish pig population, by imported spray-dried porcine plasma (SDPP). The model was based on information from literature and Danish feed companies. Moreover testing the batch of raw blood (before the spray-drying) was considered as potential risk mitigation measure in the future.

**Results:**

The median *PPlasma* was 0.2 % (90 % P.I.: 0.003 %; 2.6 %). Hence, the annual probability of introducing PEDV into the Danish pig population by imported SDPP appeared very low, and on average at least one introduction each 500 years – corresponding to 1/0.002 - could be expected. However, if PEDV survived the spray-drying process and storage was insufficient to completely remove the remaining viable virus (e.g. due to storage at low environmental temperatures during a short time period) the *PPlasma* was 4.7 % (0.06 %; 57.4 %). In that case, on average, at least one PEDV introduction each 21 years could be expected. This probability could be reduced to 0.3 % (0.004 %; 6.0 %) if the raw batch of blood could be tested before drying (corresponding to at least one introduction each 333 years on average).

**Conclusions:**

This study provides preliminary and important information on the probability of introducing PEDV into the Danish pig population by use of SDPP. Currently PED is not a notifiable disease in the EU and uncertainty was present in our estimates due to possible underreporting in EU Member States, from which SDPP is imported into Denmark. In the future, PED might become a notifiable disease, and in such a case, new knowledge could become available on its epidemiology. Moreover, SDPP could be imported more safely if: producers find a way to substantiate freedom from disease (at least) in herds delivering blood for SDPP, the batch of blood tests negative for PEDV and conditions for processing/storage required by the international laws are respected.

## Background

Porcine epidemic diarrhea (PED) is a pig disease caused by an RNA virus (PEDV), which belongs to the *Alphacoronavirus* genus of the Coronaviridae Family [1–4]. PEDV was first reported in the 1970s in the UK [3, 5] and it has since then been found in some EU Member States, parts of Asia and in the Americas. The outbreaks observed in the 1970s [6], were not as devastating in suckling pigs as those seen today. For detailed reviews on the pathogenesis and epidemiology of the disease, and its distribution worldwide we refer to Song and Park [2], EFSA [3] and Martelli et al. [7].

Domestic pigs are the only known hosts and the occurrence of the disease in other animal species is unknown [3]. PED can be considered a re-emerging disease though it is currently not included in the OIE List of Diseases and is not a notifiable disease in the EU [3]. Nevertheless, there has been an increase in the disease notifications to the OIE’s World Animal Health Information System [4].

In Europe, in the 1990s, serological surveys made in different Member States showed a low prevalence of the virus. During the last decade a few recent outbreaks have been reported from the EU (e.g. in Italy, Germany, Belgium, The Netherlands and Ukraine) [3, 7, 8].

The PED virus strains seen today affect pigs of any age, but most severely piglets, in which morbidity and mortality can be as high as 100 % [4]. In finishers, boars and sows, morbidity could be as high as 90–100 %, while mortality ranges from 0 to 4 % [3, 4, 7, 9].

Direct transmission occurs mainly by the oral route, e.g. through ingestion of feed contaminated with the virus [10, 11]. Indirect transmission could occur through fomites e.g. contaminated trucks and equipment [3, 12].

**Fig. 1:**
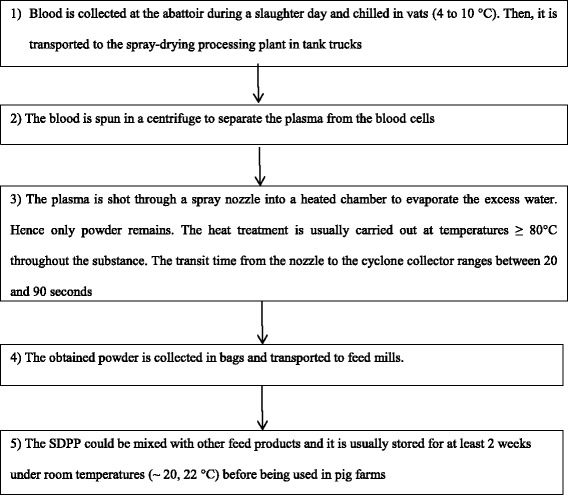
SDPP producing diagram. N.B. Here we give a general diagram, and as stated by Sampedro et al. [20], there are several parameters that could vary between spray dryers (e.g. the flow rate inputs, the retention time etc.). Those parameters could affect the survivability of PEDV during the spray-drying process [3, 20]

Pig blood products, such as spray-dried porcine plasma (SDPP), which can be fed to piglets as a feed supplement, have been suspected as a possible route of virus spread [3, 11]. However, some studies suggested that SDPP is not a likely source of infectious virus [13, 14]. In fact, for the spray-drying process (Fig. 1), efficient combinations of temperature and time should be used. Usually the temperature is ≥ 80 °C throughout the substance and the plasma transit time in the dryer is between 20 and 90 s [15–20]. Nevertheless, it would be helpful to clarify exactly which combinations of temperature and time are needed to inactivate PEDV.

The incubation period of the disease is between 1 and 4 days. The infectious period can last between 1 and 5 weeks [4, 21, 22], and growing pigs can recover within 1 or 2 weeks. Maternal antibodies can protect piglets and re-infection might occur after immunity wanes [4, 6, 7, 23].

Hence, PEDV is capable of causing large-scale outbreaks of diarrhea within infected herds [7, 9]. From a clinical point of view, the disease is similar to other forms of gastroenteritis because symptoms include anorexia, vomiting, watery diarrhea and dehydration followed by weight loss. The specific clinical signs depend on the age of the animals. Usually, the lesions found at autopsy include thinning of the intestines and atrophic enteritis [3, 4, 9].

Differential diagnosis must be made with a) transmissible gastroenteritis (TGE) [24], which is caused by a similar virus (TGEV) of the Coronaviridae Family, b) bacteria (*Clostridium spp., E. coli,* etc.) and c) parasites (*Isospora* suis, *Cryptosporidium* spp., nematodes, etc.) which can cause somewhat similar symptoms [4]. Samples of fresh feces, small intestine and serum can be tested for diagnosis. The agent can be identified by RT-PCR, ELISA, Immunohistochemistry (IHC), and virus isolation [2, 4, 7, 11, 22, 24, 25].

Vaccines have been developed in Asian countries [2], but they are currently not in use in Europe to our knowledge [3] and there are no specific treatments for the disease. Hence, prevention strategies focus on biosecurity. All-in-all-out production systems can be efficient to control PED in countries with endemic status [4].

PEDV has never been reported in Denmark [3, 26] and keeping the free status of Danish pig herds is a priority. There is almost no import of live pigs into Denmark, neither for breeding nor for slaughter. Therefore, other ways of disease introduction are of concern. The aim of the present study was to investigate to which extent imported SDPP could represent a source of PEDV infection for Danish pig herds. Thus, we assessed the annual probability (*PPlasma*) of introducing PEDV into the Danish pig population through imported SDPP. The potential effect of testing raw blood batches before spray-drying was considered as a potential risk mitigation measure although this would depend on the development of a highly sensitive PCR test. Finally, sources of uncertainty around the *PPlasma* estimate were identified, which can be useful for prioritization of new studies on PEDV.

## Results

### Probability that a batch of blood collected during a slaughtering day is contaminated

The median probability (*PContBatch*) that a batch of blood collected at the abattoir abroad is contaminated with PEDV, due to the slaughtering of at least one infected pig, was 6.0 % (90 % Prediction Interval: 0.3 %; 22.6 %).

### Annual probability of PEDV introduction into Danish pig herds by imports of SDPP

According to the reference simulation scenario (Table 1, I), the median annual probability (*PPlasma*) of introducing PEDV in at least one Danish herd (with at least one piglet infected due to SDPP) was 0.2 % (90 % P.I.: 0.003 %; 2.6 %). Hence, the *PPlasma* appeared very low and on average at least one introduction each 500 years – corresponding to 1/0.002 - could be expected.

**Table 1:** Alternative scenario analysis. Annual probability (*PPlasma*) of PEDV introduction into the Danish pig population

Simulation scenario^a^	5^th^ Percentile	Median	Years^b^	95^th^ Percentile
Scenario I (reference)	0.003 %	0.2 %	500	2.6 %
Scenario II (*PSurvDry* = RiskUniform [(0; RiskUniform (5.5 %; 60 %)]	0.06 %	4.7 %	21	57.4 %
Scenario III (Rations* 2)	0.0055 %	0.40 %	250	5.28 %
Scenario IV (PInf* 2)	0.0056 %	0.37 %	270	5.30 %

^a,^ *PSurvDry* = probability that PEDV survives to the spray drying according to Sampedro et al. [20] and assuming probability of virus survival during storage 100 %; *Rations* = annual number of individual SDPP doses used in Denmark, *PInf* = probability that a naïve piglet fed with SDPP, where viable PEDV is present in traces, becomes infected

^b^In the column “years” we report how often at least one introduction of PEDV into the Danish pig population could be expected, according to the estimated median *PPlasma*

### Output of alternative scenario analysis

In the alternative scenario analysis (Table 1), setting the probability of incomplete removal of viable PEDV during storage (after drying) to 100 % (Table 1, scenario II), caused a remarkable increase in the *PPlasma* compared to the reference scenario (Table 1, scenario I). In fact, the median *PPlasma* increased from 0.2 % in scenario I, to 4.7 % (90 % P.I.: 0.06 %; 57.4 %) in scenario II. In the latter case, at least one PEDV introduction each 21 years could be expected (1667; 2).

Doubling the number of imported SDPP rations (Table 1, scenario III), or doubling the probability of infection at the single animal level (Table 1, scenario IV), lead to twice as high a probability compared to scenario I, but still the annual *PPlasma* appeared to be very low (median around 0.4 %).

### Effect of testing the raw batch of blood

We found that if the batch of blood collected at the abattoir could be tested before the spray-drying process, the *PPlasma* of scenario II (where storage is not efficient for complete removal of all the remained viable PEDV) would be reduced from 4.7 to 0.3 % (90 % P.I.: 0.004 %; 6.0 %). The latter would correspond to at least 1 introduction each 333 years, on average.

## Discussion

### Annual probability of PEDV introduction into the Danish pig population related to imported SDPP (current situation)

Spray-dried plasma proteins are considered a high-quality nutrient for weaning piglets and they can have several beneficial effects on piglet’s health [27]. Nevertheless, during the recent years concern has arisen on the probability of transmitting swine diseases such as PED, through the use of SDPP [3, 11, 28].

The present study was carried out to assess the annual probability of PEDV introduction into the Danish pig population by imported SDPP, at a time with limited knowledge about the virus. It gives important information for farmers and risk managers. Additionally, this work highlights the importance of the SDPP producing and storage steps, where new studies could be prioritized to reduce uncertainty on the estimated *PPlasma* (e.g. during short storage in winter). Moreover, the developed stochastic simulation model could be used in other countries easily, by adjusting the herd incidence (Eq. 1, *HerdInc*) in SDPP exporting countries (e.g. if SDPP is imported from non-EU countries) and the annual number of imported SDPP rations (Eqs. 2–3, *Rations*).

We assumed that blood sera batches used to produce SDPP could be contaminated with PEDV. In that case - and based on available data - the probability of PEDV introduction to Denmark would be higher than 0. Nevertheless, if the storage period and the storage temperatures were adequate to completely inactivate viable PEDV from contaminated SDPP, the *PPLasma* appeared to be very low. On average, it corresponded to one introduction each 500 years which may be interpreted as a negligible risk.

The input where we had the largest lack of knowledge was the probability of infection after exposure to contaminated SDPP at the individual piglet level (*PInf*). We considered it adequate to account for uncertainty by setting the *PInf* in a range of values between 0 and 1/1,000,000; to reflect the debate whether low levels of virus are viable for causing infection in weaning pigs fed with SDPP [3, 11, 13, 14]. Sampedro et al. [20] estimated that, although in rare situations (worst case scenario) viable virus could remain in SDPP, even after storage. Hence, in absence of precise data - and observation of devastating outbreaks - we make use of the precautionary principle. We find that the values used for *PInf* can be considered fairly low and fairly high at the same time, with the current knowledge we have at hand. In fact, it seems that the probability of infection at the single animal/herd level should be very low (possibly close to 0) [13, 14]. Nevertheless, at country-level, such a low probability may still lead to disease introduction, because millions of rations of SDPP are used (in Denmark approximately 14.3 millions per year).

In previous experimental studies, where *PInf* has been investigated with live pigs, a low number of animals have been used e.g. [13, 14] due to obvious animal welfare concerns and costs. Therefore, the outputs of the risk assessment we have carried out, are difficult (if not impossible) to validate at the experimental level, since it is impossible to undertake studies, where millions of pigs are purposely fed with contaminated SDPP rations for several years. This is in line with EFSA who suggested that testing a large number of animals is required, to detect low concentrations of infectious PEDV in SDPP, and thus, the probability of detecting at least one infected piglet during a bioassay would be low [3].

Hence, in the reference scenario (Table 1, I) we set the maximum *PInf* = 1/1,000,000 and we used a Beta distribution to take into account the uncertainty around such a value. This may correspond to a negligible individual risk of infection, but still higher than 0 (hence infection seems unlikely but not impossible).

Furthermore, we assessed the probability of PEDV introduction at national level by using the variable *“Rations”* in Eqs. 2–3, to estimate the *PPlasma*. If in the future, the quantity of imported SDPP increases remarkably (e.g. more than double), the model presented in this study could be easily re-run to assess the *PPlasma* with the adjusted number of *Rations*.

### Information from alternative scenario analysis

According to the alternative scenario analysis, the input with the highest impact on the estimated *PPlasma* was the probability that any virus, which may have survived the processing, also survives the storage. In scenario II (Table 1), we set that probability to 100 %. Here, we showed that if PEDV is not completely inactivated by the spray-drying process and the storage is not effective to eliminate all remaining viable virus (e.g. during winter), at least one PEDV introduction each 21 years could be expected on average, while according to the upper limit of the 90 % PI, at least one introduction each 2 years could be expected. Nevertheless, the latter output was a rare result since a *PIntro* ≥ 57.4 % occurred in only 5 % of the 10,000 iterations we used.

Hence, based on results of scenario II, it must be taken into account that temperatures and storage time could vary between SDPP producing countries/seasons. Dee et al. [29] demonstrated that PEDV can remain viable for extended periods (e.g. up to 45 and 180 days in stored complete feed and soya bean meal, respectively), and that the survival of the virus is ingredient-dependent.

In Europe, the SDPP imported from countries outside the EU should be stored for at least 6 weeks in dry warehouse conditions under room temperature [3, 16]. For EU Member States storage periods have not been defined. The survival of PEDV in a final feed product can be affected by several parameters such as: temperature, time and relative humidity. For instance, in a recent study, Goyal reported that (e.g. at 4 and −20 °C) the virus could survive up to 4 weeks (last time when the samples were tested) in slurry and wet feed; and could cause infection at a very low infectious dose (dilution of 10^−8^) in experimentally treated pigs [30].

Sampedro et al. suggested that further research should be carried out to verify and refine the required time and temperature conditions of storage, particularly in cold climates [20]. Accordingly, further experimental studies on the survivability of PEDV at very low temperatures (e.g. around 0 °C in winter) during different storage periods (shorter or longer than 6 weeks) would be beneficial to reduce the uncertainty related to our *PPlasma* estimates. In the current situation, agreements between trading partners could be made regarding the best storage periods to use, provided that the minimum requirements of the international legislation [3, 15, 16, 18] are followed.

Regarding the quantity of imported SDPP used in Danish pig herds, a double amount should not pose a high risk, under the assumption that both the spray-drying and the storage phases occurred in optimal conditions (Table 1, scenario III).

The change caused in the *PPlasma* by increasing the *PInf* was relatively high (Table 1, scenario IV), though the *PPlasma* remained <1 %. Hence, our most uncertain parameter had low impact on the results.

### Impact of testing raw blood batches as a risk mitigation measure

SDPP producers should be encouraged to find a way to substantiate freedom from infection at least in the herds delivering blood for SDPP. If a highly sensitive PCR test were applied on every blood batch, then only test-negative batches could be used to produce SDPP destined for pig feed. In this way, the *PPlasma* could be reduced to very low levels, as we showed for the situation where SDPP is stored under non-optimal conditions. By applying raw blood testing and good SDPP storage, the risk of spreading PEDV between countries by imported SDPP could become negligible.

Further risk-mitigating action (s) could consist of using liquid (e.g. formaldehyde-based) antimicrobials in feed [29], and/or importing SDPP only from countries where freedom from PED is substantiated with high confidence (e.g. by testing a sample of blood specimens from a sufficient number of animals/herds, randomly selected). In such a case, the inputs of herd incidence (*HerdInc*) and within-herd prevalence (*WHP*) used in this study would be around zero and the probability of PEDV introduction to Denmark could become negligible (due to low *PContBatch*, Eq. 1). Therefore, if in the future, PED becomes a notifiable disease in EU, the infection status of Member States (or herds within Member States) could be defined with less uncertainty. Consequently, risk assessments at national level would become more precise because monitoring of outbreaks would increase (more precise *HerdInc* and *WHP* inputs would become available). As suggested by EFSA [3] “additional sequence data are required to understand PEDV evolution in Europe and the possible link with PEDV strains circulating in other parts of the world”. Currently (mid-2015) PED is not a notifiable disease, and it is considered questionable whether it is feasible to substantiate country freedom from disease, as it would require a costly surveillance program.

Thus, our estimates could be seen as preliminary results, which can be updated (and can become more precise) when 1) further knowledge on the epidemiology of PED becomes available, and/or 2) PED becomes notifiable.

With the current knowledge we have at hand, we could not exclude that SDPP produced from a contaminated batch of blood could carry viable PEDV. For that reason we carried out the risk assessment.

### Limitations and suggestions for further studies

In this study, as in any risk assessment, some limitations were present. This was due mainly to the fact that, PED is a rare disease in the EU and most of the information we found in literature comes from a few recent studies. Most of those were carried out during the last decade, and thus, the current information on the used parameters is scarce. As pointed out by Sampedro et al. [20] there is paucity of data on the thermal inactivation of PEDV and there is limited understanding on the mechanisms of the inactivation of viruses during the spray-drying processes. Hence, in our risk assessment, inputs were set by taking into account as much as possible the information we found in previous scientific studies and uncertainty was included in all inputs.

Moreover, risk assessment studies can be considered as progressive processes, which can give important information for decision-makers. This information can be used to prioritize new studies so that uncertainty on the estimates can be reduced.

For instance, we assumed that the blood was collected at the abattoir through a closed draining system. If this is not the case, and if cross-contamination can occur, then the probability that a batch of plasma contains PEDV (*PContBatch*) could be higher than we estimated. This would be the case when an open blood draining system is used at the abattoir (blood collected into buckets or trays). Nevertheless, we assumed that usually, in EU abattoirs where thousands pigs are slaughtered a closed system is used, with a hollow knife connected directly to the vacuum piping [31]. In countries with endemic PED, new studies could be carried out to investigate the different probabilities of contaminating the blood batch if an open vs. a closed draining system is used.

Furthermore, we assumed that if the batch of blood was contaminated with PEDV, the virus could remain viable in the SDPP after the drying process and the storage, with a probability between 0 % and 1.1 % (*PSurvDryStor* in Eq. 2). As explained above, this assumption was based on the simulation output by Sampedro et al. [20]. On the other hand, survival could vary and could be affected by the different steps of the SDPP process and by the dryer used, as suggested by EFSA [3].

Testing of pooled blood before spray-drying could be a solution. At the same time, it must be remarked that when we investigated the importance of testing raw blood, we assumed that the sensitivity of such a PCR test was similar to that reported by Song et al. [32] for testing feces and intestinal samples. The amount of PEDV RNA detected in serum is usually lower compared to the levels in feces [33]. Hence, new studies should investigate the sensitivity of the PCR when used on samples of blood pooled from several thousand animals. Eventual effects of dilution and interactions between PEDV and antibodies (eventually present in the batch of blood) should be studied as well.

Further studies on the relation between prevalence of animals which shed PEDV in feces and prevalence of viremic pigs (which have PEDV in blood) would be beneficial, since updated estimates would become available for the within-herd prevalence input (*WHP* in Eq. 1).

Finally, in our study, we did not consider the possibility of SDPP cross-contamination after the spray-drying, e.g. during storage, transport and/or in the farm. We assumed that usually, good manufacturing practices are followed by producers and good biosecurity measures are applied by transporters and farmers. Further studies could investigate the probability of cross-contamination of SDPP after processing.

## Conclusions

The present work gives important information on the probability of introducing PEDV into the Danish pig population through the feeding of SDPP to naïve Danish weaning piglets. The median annual probability of disease introduction appeared to be very low if the drying and storage phases were made under strict and efficient biosecurity measures. However, it appeared that the probability of introduction could increase dramatically if the processing is not fully efficient to eliminate the virus and the SDPP is stored under cold conditions and for a short time period. That probability could be reduced if SDPP producers find a way to substantiate freedom from PEDV at least in the herds delivering blood for producing SDPP. Alternatively, producers could test raw blood batches prior to processing. At the same time, producers should provide documentation that the international legislation, regarding the different phases of SDPP processing and storage, has been complied with. Finally, to reduce uncertainty, further studies could be carried out on 1) the sensitivity of tests, which can be used on raw blood batches and 2) the survivability of the PEDV during short periods of SDPP storage at very low temperatures (e.g. during winter).

## Methods

To assess the annual probability (*PPlasma*) that at least one Danish piglet being fed imported, contaminated SDPP becomes infected with PEDV, we set up and used a stochastic simulation model. Different variables were taken into account, according to the diagram reported in Fig. 1 (representing the SDPP producing layout), and using the information we found in literature. Fifteen Danish feed producing companies were consulted. They provided information about the volume and origin of SDPP imported to Denmark during a 1-year period.

The probability that a matrix (in our case SDPP fed to naïve piglets) transmits the infection to animals depends on (1) the probability that the matrix is contaminated with PEDV and (2) the probability that an exposure to such a matrix leads to infection in a susceptible pig [3]. Thus, before assessing the *PPlasma,* we investigated the probability (*PContBatch*) that a batch of blood collected at the abattoir (abroad) is contaminated with PEDV. If contamination at the abattoir-level is impossible, there is no need to estimate the *PPlasma*. Otherwise, the *PPlasma* needs to be estimated.

An alternative scenario analysis was subsequently undertaken, to investigate the impact of the main inputs on the *PPlasma*. According to the output of this analysis, we also investigated the impact of testing for PEDV the batch of raw blood collected at the abattoir, as a risk mitigation measure.

The model was developed in @Risk 6 (Palisade Corporation). Runs were made using 10,000 iterations and Latin hypercube.

### Probability that the batch of blood is contaminated with PEDV

**Fig. 2:**
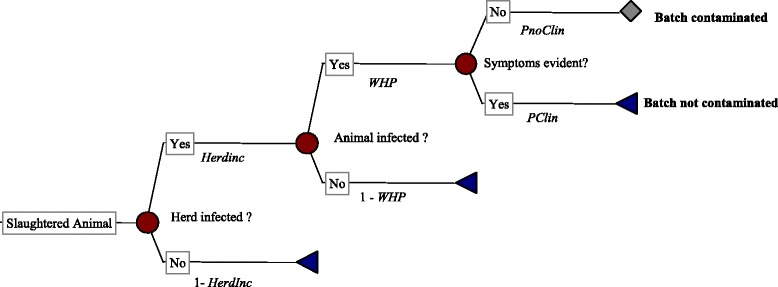
Stochastic scenario tree to assess the probability (*PContBatch*) that at least one slaughtered pig is infected. *Herdinc* = incidence of infected herds abroad, *WHP* = within-herd prevalence, *PnoClin* = probability that the infected pig is slaughtered since it does not show symptoms of infection before slaughter at ante-mortem inspection. The latter is given by 1 – *PClin,* where *PClin* represents the probability that the infected pig shows symptoms (e.g. watery diarrhea) and so it is not slaughtered for human consumption

The probability that a batch of blood is contaminated with PEDV (at the abattoir-level) was estimated using the stochastic scenario tree reported in Fig. 2 and Eq. 1:

1 $$ PContBatch=1-{\left[1-\left( HerdInc*WHP* PnoClin\right)\right]}^{\wedge Npigs} $$

Where, *PContBatch* represents the probability that at least one infected animal contributes to the batch of blood collected during a slaughtering day. In such a case we assumed that the virus from the individual animal (s) was homogeneously distributed within the blood vat. Moreover, we assumed that the blood was usually collected through a closed draining system [3, 31].

*HerdInc* is the incidence of PEDV infected herds in the SDPP producing country, and *WHP* is the within-herd prevalence in infected herds. Thus, *HerdInc* represents the probability that the herd sending pigs for slaughtering is infected, while *WHP* is the probability that the slaughtered pig, from an infected herd, is actually infected.

*PnoClin* is the probability that an infected slaughter animal does not show symptoms (e.g. diarrhea and vomiting), so that the farmer sends the animal to slaughter and the ante-mortem inspector allows the slaughtering.

*Npigs* is the daily number of slaughtered pigs (in a single abattoir abroad), which contribute to a batch of blood.

### Herd incidence in SDPP producing countries from where pig plasma is imported *(HerdInc)*

According to a recent report from EFSA [3], the incidence of PEDV infected herds (*HerdInc*) in EU Member States is expected to be low.

Based on the information obtained from Danish feed producing companies and from the Danish Agriculture & Food Council, SDPP is imported from Spain, Ireland, United Kingdom, Germany, The Netherlands and Poland.

Since blood is usually processed soon after collection at the slaughterhouse, we assumed that the imported SDPP came from the country, where the pigs were slaughtered (e.g. if produced in Spain, only Spanish pigs contributed to a daily batch of blood). Hence, in our model, *HerdInc* represented the annual incidence (new cases) of PEDV infected herds in an EU Member State.

The only well-documented epidemic of PEDV in Europe (with 63 infected herds) occurred in northern Italy, between May 2005 and June 2006 [3, 7]. During this time period, the Italian pig population was composed of approximately 101,000 pig herds [34].

On the other hand, most of the EU Member States have no active monitoring on this disease in place, and consequently underreporting could be present [3]. Therefore, we decided to set *HerdInc* as Uniform distribution from 0 (no infected herds in the SDPP producing country), to 63 [3, 7] infected herds out of 101,000 pig herds present in Italy [34] during a 1-year period. To include in the *HerdInc* the uncertainty due to eventual under-reporting and lack of knowledge, we set the maximum value as a Beta distribution (*s* + 1; *n-s* + 1), where *s* is the annual number of infected herds found in Italy (63 in our case) and *n* is the overall number of herds present in the same country (101,000).

### Within-herd prevalence (*WHP*)

The prevalence of infected animals within finishers and/or sows herds (*WHP*) was set according to findings from Pijpers et al. [23]. To represent the variation in the within-herd prevalence, we set *WHP* with a Pert distribution (RiskPert) ranging from 46 to 100 % and with mode equal to the median of the values (Table 2) reported by Pijpers et al. [23]. Often suckling pigs, pregnant sows, 3 to 10 week-old piglets and fattening pigs are housed in separate facilities [23]. Accordingly, we assumed that the proportion of animals shedding virus (in feces) within each animal group, could represent the *WHP* in different kind of pig herds. Moreover, from Pijpers et al. [23], we only used data on prevalence of infected animals, older than 10 weeks (Table, 2), since usually those are sent to slaughter where blood is collected for producing SDPP.

**Table 2:** Inputs used for the within-herd prevalence of viremic animals (*WHP*), based on Pijpers et al. [23]

Animal group	Proportion of infected animals between those tested per group
Pregnant sows^a^	4/6 = 67 %
Farrowing sows^a^	2/3 = 67 %
Fattening pigs^a^	6/6 = 100 %
Pregnant sows^b^	7/8 = 88 %
Fatttening pigs^b^	6/13 = 46 %
*WHP* distribution	RiskPert (46 %; mode = median of all values; 100 %)

^a^Animals tested during the first month of the outbreak

^b^Animals tested during the 10 months following the beginning of the outbreak

N.B. According to Pijpers et al. [23], we used the proportion of animals shedding PEDV in feces within groups of pigs older than 10 weeks, since only those animals were assumed to contribute to the blood batch collected at the abattoir

To date, very few studies investigated viremia in PEDV infected pigs. In Pijpers et al. [23], animals were tested for PEDV presence in feces. We assumed that the proportion of animals shedding PEDV in feces (in each age group) corresponded to the within-herd prevalence of viremic animals (with PEDV in serum).

It is usually assumed that viremia can last for short periods. Gerber et al. [13] did not detect PEDV in plasma of infected pigs at peak of disease. On the other hand, our assumption is supported by other studies. In fact, Hesse et al. [21] detected viremia in infected pigs and suggested that there should be a correlation between viremia and virus shedding (either fecal or nasal), while Jung et al. [33] reported that severe diarrhea, vomiting and fecal shedding may be accompanied by viremia. The latter authors detected viral RNA in serum of infected pigs, with titers between 4.8 and 7.6 log_10_ GE/mL.

### Variables for slaughtered pigs contributing to a batch of blood (*PnoClin* and *Npigs*)

We assumed that in a naive pig population (as should be the case in most European pig populations) fatteners and adults can show a morbidity of up 90–100 %, with the typical clinical signs of PED such as watery diarrhea [3, 9]. We set the probability that an adult infected pig shows symptoms as *PClin* = RiskUniform (90 %; 100 %), while the probability of not showing symptoms (in Eq. 1) was *PnoClin* = 1- *PClin.*

Moreover, a batch of blood, which can be used to produce SDPP, is usually composed of blood pooled from 6,000 to 10,000 pigs slaughtered on the same day [13]. In our model, we set this information as a uniform distribution where *Npigs* = RiskUniform (6,000; 10,000).

### If complete virus removal is not possible, can a piglet become infected by being exposed to a SDPP contaminated ration?

According to Opriessnig et al., RNA of PEDV could be found in traces in commercial SDPP, but it should not cause infection in animals [14]. In contrast, Pasick et al. argued that contaminated SDPP could cause PED outbreaks [11]. Those authors reported that in January 2014, the first PED case in Canada was confirmed in a swine herd in south-western Ontario. Several lots of feed and SDPP imported from the USA tested positive for PEDV by RT-PCR. Accordingly, it was suspected that the contaminated feed/SDPP may have caused PEDV introduction into Canada. Three of the PEDV-positive SDPP samples were orally inoculated to 12 (3-week-old) piglets confined in the same barn. All the animals became infected (with positive rectal swabs), suggesting that at least one of the contaminated SDPP samples contained viable PEDV. Sampedro et al. estimated by a simulation study that PEDV could remain viable in processed positive blood batches [20].

Thus, currently, there is debate around the world, on the viability of PEDV eventually present in processed (spray-dried) contaminated plasma. It is in fact argued that RNA could be present in a ration of SDPP, but such a low amount of virus should not be able to cause disease in the animal (e.g. the virus or parts of it are present, but it has been killed by the spray-drying process and/or by the storage time) [13, 14].

For these reasons, to take into account for the different points of view we found in the most recent literature [3, 11, 13, 14, 17, 20], we set the probability that a weaning piglet becomes infected by receiving a contaminated ration of SDPP as *PInf* = RiskUniform [0; RiskBeta (1 + 1; 1,000,000-1 + 1] within Eqs. 2–3 (below). Therefore, here “contaminated” means that viable PEDV could be present in traces, and we assumed uncertainty on the probability that such a virus could cause infection (this uncertainty is represented in the *PInf* range). In *PInf* the minimum represents the situation where it is impossible that the piglet becomes infected (since all virus is non-viable), while the maximum represents a negligible individual risk of infection by consumption of a SDPP ration contaminated with low levels of viable virus. For the maximum, a Beta distribution was used to represent uncertainty.

### Probability of PEDV introduction into Danish pig herds

The annual probability (*PPlasma*) that at least one Danish piglet becomes infected with PEDV due to imported contaminated SDPP was estimated as:

2 $$ PPlasma=1-{\left[1-\left( PContBatch* PSurvDryStor* PInf\right)\right]}^{\wedge Rations} $$

*PSurvDryStor* was the probability that PEDV present in the contaminated raw batch of blood survived to both the spray-drying process and the storage period. We set this input according to the information from Sampedro et al. [20]. The latter authors carried out a risk assessment and simulated that between 0 and 1.1 % (worst case scenario) of the positive raw blood batches could remain positive with viable PEDV, after the drying process and storage at 20, 22 °C for 2 weeks. Hence in our model, we set *PSurvDryStor* = RiskUniform (0 %; 1.1 %).

Moreover, we assumed that if a batch of SDPP was still contaminated with viable PEDV, all the derived rations of SDPP were contaminated homogeneously with PEDV present in traces.

The term *Rations* in Eq. 2 is the number of SDPP rations used in Denmark during a 1-year period. Annually, 250 t of SDPP are imported to Denmark (unpublished data from the Danish feed companies). The amount of SDPP used in a ration of feed for a weaning piglet, ranges between 4 and 5 % corresponding to approximately 15–20 g of SDPP per piglet per day. Hence, we set *Rations* = 250,000,000 g / RiskUniform (15; 20) g ~ 14.3 million rations used per year.

### Alternative scenario analysis

In this section we carried out an alternative scenario analysis (what-if analysis) to assess the *PPlasma* under circumstances different from those assumed in the previous sections.

For that purpose, we compared the *PPlasma* estimated with the reference simulation scenario described above (Table 1, Scenario I) with other scenarios obtained by a) setting the probability of PEDV survival during the SDPP storage equal to 100 % (Table 1, Scenario II), b) doubling the annual number of SDPP imported rations (Table 1, Scenario III), and c) doubling the probability that a susceptible Danish naïve piglet fed with SDPP contaminated with PEDV becomes infected (Table 1, Scenario IV).

We investigated scenarios from II to IV for different purposes. For instance, in the reference scenario (Table 1, I), *PSurvDryStor* was set according to simulation estimates from Sampedro et al. [20], who assumed SDPP storage at high temperatures for a few weeks. PEDV is known to be more resistant in cold and wet conditions than at high temperatures [3, 17, 19, 30]. Hence, in scenario II we used the conservative assumption that a short storage period (e.g. of 2 weeks) with low temperatures (e.g. during winter) is not completely efficient to inactivate all viable virus, which has eventually survived to the spray-drying process. Additionally, as in Sampedro et al. [20] we assumed that the virus surviving the spray-drying can be infectious. Thus, in scenario II, only the spray-drying phase was considered as a potential PEDV inactivation step and we set the probability that the virus survived such a step as *PSurvDry =* RiskUniform [(0; RiskUniform (5.5 %; 60 %)]. The distribution used for the maximum limit was still taken from Sampedro et al., who estimated that up to 5.5–60 % of the positive raw blood batches could remain positive with viable virus after the spray–drying [20]. Still, we assumed that the remaining PEDV was homogeneously present in all the SDPP rations derived from a contaminated blood batch.

With scenario III, we investigated the impact of an eventual increase in the annual number of imported SDPP rations (used in Danish pig herds).

With scenario IV, we investigated the impact of using a maximum limit for *PInf* higher than negligible (maximum > 1/1,000,000).

### Impact of testing raw blood batches with PCR, as a risk mitigation measure

In the alternative scenario analysis we found that if SDPP is contaminated with PEDV and storage does not allow complete elimination of all remaining viable virus, there could be a remarkable increase in the *PPlasma* (Table 1, Scenario II).

The investigation of critical control points in the SDPP producing layout was out of the scope of this article. Nevertheless, under a situation of inefficient storage, we investigated the opportunity of testing the raw batch of blood as a risk mitigation measure.

For this purpose, a single PCR testing could be carried out on the blood pooled from several thousand pigs (*Npigs* in Eq. 1) slaughtered in a day. As an example, we considered a PCR with sensitivity (*Se*) similar to that reported by Song et al. [32], for testing fecal and intestinal samples (*Se* = 92.9 %). If the batch of blood is negative, then *PInfBatch* should be around zero. As a consequence, also *PPlasma* could become very low (according to Eq. 3). In contrast, if the virus is detected at this stage, the batch of blood could be destined to other purposes or for other animal species.

Therefore to assess the impact of testing raw blood batches we estimated *PPlasma* by:

3 $$ PPlasma=1-{\left[1-\left( PContBatch* PFalseNegBatch* PSurvDry* PInf\right)\right]}^{\wedge Rations} $$

Where, *PSurvDry* replaced *PSurvDryStor* used in Eq. 2 (as in Table 1, Scenario II)*.* Moreover we introduced the input *PFalseNegBatch,* which represented the probability that a batch of blood containing viable PEDV, from at least one infected animal (out of the *Npigs* slaughtered daily) is classified as false negative by the PCR used. Thus, *PFalseNegBatch* was given by 100 – *Se.*
